# Genome investigation suggests *MdSHN3*, an APETALA2-domain transcription factor gene, to be a positive regulator of apple fruit cuticle formation and an inhibitor of russet development

**DOI:** 10.1093/jxb/erv366

**Published:** 2015-07-28

**Authors:** Justin Lashbrooke, Asaph Aharoni, Fabrizio Costa

**Affiliations:** ^1^Research and Innovation Centre, Fondazione Edmund Mach, 38010 San Michele all’Adige (Trento), Italy; ^2^Department of Plant Sciences, Weizmann Institute of Science, PO Box 26, Rehovot 76100, Israel; ^3^Institute for Wine Biotechnology, Stellenbosch University, Stellenbosch 7602, South Africa

**Keywords:** APETALA2-domain, apple, cuticle, fruit, MdSHN3, QTL, russet.

## Abstract

The outer epidermal layer of apple fruit is covered by a protective cuticle. Composed of a polymerized cutin matrix embedded with waxes, the cuticle is a natural waterproof barrier and protects against several abiotic and biotic stresses. In terms of apple production, the cuticle is essential to maintain long post-harvest storage, while severe failure of the cuticle can result in the formation of a disorder known as russet. Apple russet results from micro-cracking of the cuticle and the formation of a corky suberized layer. This is typically an undesirable consumer trait, and negatively impacts the post-harvest storage of apples. In order to identify genetic factors controlling cuticle biosynthesis (and thus preventing russet) in apple, a quantitative trait locus (QTL) mapping survey was performed on a full-sib population. Two genomic regions located on chromosomes 2 and 15 that could be associated with russeting were identified. Apples with compromised cuticles were identified through a novel and high-throughput tensile analysis of the skin, while histological analysis confirmed cuticle failure in a subset of the progeny. Additional genomic investigation of the determined QTL regions identified a set of underlying genes involved in cuticle biosynthesis. Candidate gene expression profiling by quantitative real-time PCR on a subset of the progeny highlighted the specific expression pattern of a *SHN1*/*WIN1* transcription factor gene (termed *MdSHN3*) on chromosome 15. Orthologues of *SHN1*/*WIN1* have been previously shown to regulate cuticle formation in *Arabidopsis*, tomato, and barley. The *MdSHN3* transcription factor gene displayed extremely low expression in lines with improper cuticle formation, suggesting it to be a fundamental regulator of cuticle biosynthesis in apple fruit.

## Highlight

QTL analysis of an apple population identifies genomic regions linked to cuticle formation and subsequent russet formation. Further investigation suggests that *MdSHN3* is a major regulator of cuticle biosynthesis in apple.

## Introduction

The plant cuticle is a continuous lipophilic layer covering the above-ground epidermal layer of the plant. While the cuticle contributes to the structural support of the cell, its principal role is probably the interaction point between the plant and its environment where it contributes to the protection against both biotic and abiotic stresses (Bargel *et al.*, 2006; Yeats and Rose, 2013). In particular, the cuticle structure is highly adapted to overcome dehydration stress efficiently (McCourt *et al.*, 2004). Consisting of an ester-bonded matrix of medium chain length fatty acids embedded with very long chain fatty acids, the cuticle is extremely hydrophobic, and provides a robust barrier for movement of water and other ions (Kunst and Samuels, 2003; Pollard *et al.*, 2008; Samuels *et al.*, 2008).

The regulation of water loss is vitally important not only for the plant, but also in terms of agriculture management, since excessive water loss results in significant decreases to post-harvest fruit quality (Albert *et al.* 2013; Lara *et al.*, 2014). This is particularly true for apple (*Malus×domestica* Borkh.), a species for which its storage capacity is largely responsible for its economic success. An ability to maintain acceptable levels of water loss over an extended post-harvest can ensure fruit delivery to world-wide markets and the availability of apples year round. While mild cuticle failure may lead to excess water loss or an increase in fungal infection rates (Shi *et al.*, 2013), severe cuticle failure in fruit results in a disorder known as russeting (Lara *et al.*, 2014). This disorder results from the development of a series of microscopic cracks in the cuticle and the subsequent formation of a waterproofing periderm layer consisting largely of suberin (Khanal *et al.*, 2013). Suberin is an aliphatic polymer possessing less elasticity and less water retention qualities than cutin (Kolattukudy, 2001; Schreiber, 2010; Beisson *et al.*, 2012). A russeted surface is typically rough and corky, and is generally considered an undesirable trait for commercialization (Andre *et al.*, 2013). Apples are particularly susceptible to russet, with many naturally occurring cultivars consistently displaying this physiology, while usually non-russeted cultivars may develop russet due to environmental stresses (Knoche *et al.*, 2011; Curry, 2012). An impaired cuticular structure may also affect the mechanical strength of the apple peel, impacting handling and post-harvest processing (Saladié *et al.*, 2007). Moreover, the development of cuticular cracks also accelerates the development of flesh browning due to an enhanced oxidative process (Lara *et al.*, 2014), and may result in softer internal tissue due to the loss of an external mechanical support (Saladié *et al.*, 2005., 2007).

In light of the importance of proper cuticle formation in fruit crop production, particularly in apple, an understanding of the biosynthesis and regulation of this layer is vital. The majority of the previous research concerning these topics has been performed in tomato, and has recently been reviewed by Martin and Rose (2014) and Hen-Avivi *et al.* (2014). Investigation of the transcriptional regulation of cuticle formation in fruit suggests a complex network of transcription factors playing a role in both epidermal cell identity and cuticle formation. The WAX INDUCER1/SHINE1 (WIN1/SHN1) clade of APETELA2 (AP2)-domain transciption factors have been reported to be major factors in this network (Shi *et al.*, 2011, 2013; Hen-Avivi *et al.*, 2014). Three members of this family from *Arabidopsis* have been characterized, and have been demonstrated to act redundantly during cuticle deposition and epidermal cell patterning (Aharoni *et al.*, 2004; Broun *et al.*, 2004; Kannangara *et al.*, 2007; Shi *et al.*, 2011). On the other hand, in tomato *SlSHN3* has been identified as a positive regulator of cuticle deposition. These genes have been demonstrated to exert their influence through the downstream regulation of other transcription factors as well as cuticle biosynthesis genes (Shi *et al.*, 2013; Hen-Avivi *et al.*, 2014).

While the fruit cuticle has been largely investigated in tomato, very little is known regarding the regulatory process occurring in apple. Work presented by Albert *et al.* (2013) identified the expression profile of a number of apple genes orthologous to characterized cuticle formation genes from other species, but provided no functional information concerning the apple genes themselves. In this work, a quantitative trait locus (QTL) mapping was performed to identify genes involved in apple fruit cuticle assembly. For this purpose, a full-sib population generated by crossing ‘Golden Delicious’ and ‘Braeburn’ (‘G×B’) apple cultivars was employed, since it showed a consistent and year-stable russet segregation among seedlings, although both parental cultivars have a normal shiny skin. The subsequent *in silico* anchoring of these genomic regions on the assembled version of the apple genome (Velasco *et al.*, 2010) led to the identification of a series of genes potentially involved in cuticle formation. Candidate genes were tested via expression analysis to provide further evidence regarding their involvement in cuticle deposition. Individuals identified as having improperly formed cuticles were analysed via light microscopy and tensile testing. The results display a tight link between improper cuticle deposition and russet formation, and identified an apple orthologue to the SHN clade of transcription factors that is probably responsible for regulating fruit cuticle assembly in apple.

## Materials and methods

### Plant material and fruit russeting evaluation

To perform QTL mapping analysis oriented in the determination of the genomic regions involved in proper cuticle formation, 88 individuals from the ‘G×B’ population were selected. Within the breeding programme, this population was chosen for showing a stable and consistent development of the russeting phenotype through consecutive years. The progeny was planted originally in 2003, at the experimental orchard of the Fondazione Edmund Mach (Northern Italy), and maintained following standard technical agricultural management for pruning, crop load, and pest disease control.

Fruit for mechanical tensile testing and microscopy analysis were collected at commercial harvest, established according to standard indexes, such as skin and seed colour as well as starch accumulation (defined on a starch value of 7, based on a 1–10 scale, corresponding to the full presence of starch and absence by its complete degradation, respectively), and assessed after 2 weeks of shelf-life at room temperature. For each individual, five apples were collected and fruit russeting was initially visually inspected. The development of the suberized layer was assessed for 2 years consecutively. Fruit russeting was scored visually for its presence/absence and the estimated percentage of the fruit surface covered by russeting.

### Tensile mechanical phenotyping

The structure of the apple fruit cuticle was assessed with a novel analytical approach, making use of a TA-XT*plus* texture analyser. Data for each individual line represent repeats from five apples, from which two peel strips each were isolated. Peel strips were all cut with a width of 1cm and a length of 5.5cm. The strips were then transferred to the texture analyser (TAXT instrument, Stable MicroSystem, Godalming UK; Supplementary Fig. S1 available at *JXB* online) where they were clamped at the ends and pulled apart. The force required to stretch (and snap) the strips was recorded in relation to the distance the strips were pulled. The texture analyser instrument settings were as follows: pre-test and test speed of 1mm s^–1^, post-test speed of 5mm s^–1^, target mode distance and trigger force of 50g. The tension strength was applied until reaching the distance of 5mm. From the mechanical profiling resulting from the tensile test, five main parameters were identified through the use of an ad hoc macro compiled with the Exponent v4.0 software (provided with the instrument), and represented by gradient, maximum force, maximum force distance, area, and the linear distance of the mechanical profile (Supplementary Fig. S2; Table 1). The digital data of these parameters were then further used as phenotypic data in the final QTL mapping computation.

**Table 1.** Parameters measured during the tensile testing of apple peels

Parameter	Description	Unit
Gradient	Elasticity module (also known as Young’s module) calculated at the fixed distance established at 1mm of tension	N mm^–1^
Maximum force	Highest force value detected over the entire mechanical profile	N
Maximum force distance	Point over the *x*-axis corresponding to the maximum force value	mm
Area	Area calculated below the line of the mechanical profile	N*mm
Linear distance	Derived length of the mechanical profile	N*mm

See Supplementary Fig. S2 at *JXB* online for more detail on how each paramerter is determined.

### QTL mapping

The molecular map of this population was made within the international effort of the ‘Golden Delicious’ apple genome sequencing, in order to assemble the several contigs into scaffolds. A subset of this progeny was selected for the specific purpose of this study, considering only those individuals bearing a sufficient number of fruit. In the end, a total of 88 individuals were tested with 605 molecular markers, including simple sequence repeat (SSR) and single nucleotide polymorphism (SNP) type (for more detail, see Di Guardo *et al.*, 2013; Costa *et al.*, 2014). Markers were grouped and ordered along linkage groups using Haldane’s mapping function implemented in JoinMap 4 (Van Ooijen, 2006). Mapping parameters were set at a logarithm of odds (LOD) value of 5 and at a recombination frequency of 0.5. Marker data were integrated with phenotypic data to perform a QTL mapping analysis, computed with MapQTL 6.0 (Van Ooijen, 2009). QTL intervals were identified through the Interval Mapping algorithm, and an LOD value of 3.0 was considered as the threshold to consider a QTL as true (established after running 1000 permutations). Linkage groups and QTLs were visualized with MapChart (Voorrips, 2002) and Herry Plotter softwares (Longhi *et al.*, 2012). Each interval was further anchored and aligned on the assembled version of the apple genome.

### Light microscopy

For light microscopy, peel tissue samples were fixed and embedded in wax as described previously (Mintz-Oron *et al.*, 2008). Sections were cut to a thickness of 9 μm on an Leica 2000 microtome, and placed on glass slides. The slides were stained with Sudan IV (Buda *et al.*, 2009) and then observed with an Olympus CLSM500 microscope.

### *In silico* analysis

Nucleotide and protein sequence retrieval from the Genome Database for Rosaceae (Jung *et al.*, 2014) was performed via BLAST. Protein alignments were performed using Clustal Omega (Sievers *et al.*, 2011), and the resultant molecular phylogenetic trees were visualized using MEGA5 (Tamura *et al.*, 2011) under default parameters.

### Nucleic acid extraction and expression analysis

Apple fruits at three stages of development (early green, mature green, and harvest stage) were harvested, and the skin tissue was collected and immediately frozen in liquid nitrogen and ground into a fine powder. Total RNA extractions were performed using the RNeasy kit (QIAGEN, The Netherlands) according to the standard protocol. DNase-treated total RNA was then used to synthesize cDNA using the SuperScript VILO cDNA synthesis kit (Invitrogen, MA, USA). Quantitative real-time PCR (qRT-PCR) analysis was performed using gene-specific oligonucleotides on an ABI ViiA 7 instrument (Applied Biosystems, CT, USA) with the Fast SYBR Green Master Mix (Applied Biosystems) under default parameters. Expression was normalized using oligos amplifying a fragment of the housekeeping gene, *Md8283* (5′-CTCGTCGTCTTGTTCCCTGA-3′ and 5′-GCCTAAGGACAGGTGGTCTATG-3′). The StepOne software (Applied Biosystems) was used to generate expression data. Sequences of the gene-specific oligonucleotides are provided in Supplementary Table S1 at *JXB* online.

## Results

### Cuticle phenotyping detects differences in tensile properties

**Fig. 1.**
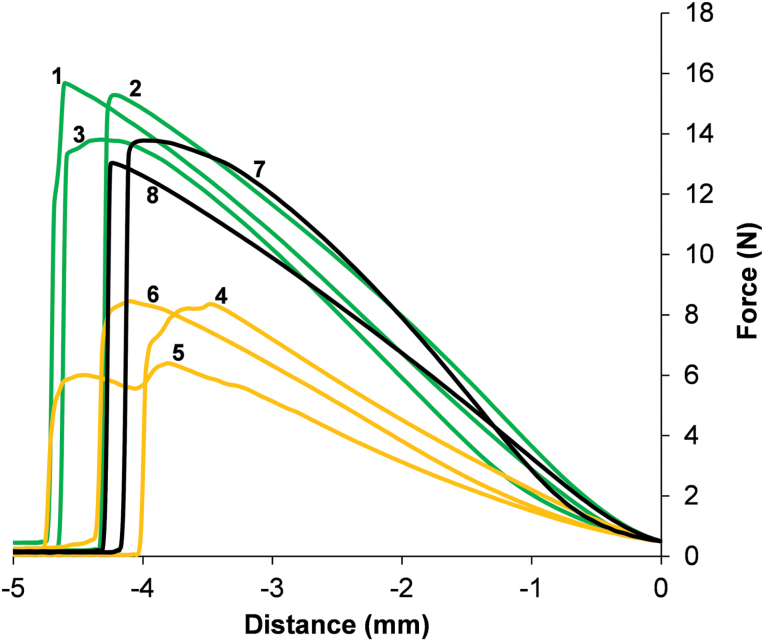
Mechanical tensile profile of apple fruit skin tissue. Mechanical tensile profiles of skin tissues from a selected subset of the population are shown. Black lines indicate parental cultivars, green lines indicate progeny deemed to have regular cuticles, and yellow lines show cultivars considered to have compromised cuticles. Specifically: 1, G×B 3; 2, G×B 6; 3, G×B 11; 4, G×B 30; 5, G×B 59; 6, G×B 90; 7, ‘Braeburn’; 8, ‘Golden Delicious’.

The development of russeting on fruit collected from each seedling of the ‘G×B’ progeny was evaluated over 2 years. For both seasons, 16 out of 88 individuals showed visual development of russeting, which occurred with different phenotypic penetrance. The surface area of fruit displaying russet formation spanned from an estimated 5% to 100%. The development of russeting, within the seedlings of the ‘G×B’ population, exhibited a 3:1 segregation ratio, supported by the statistical test of χ^2^=2.17. However, as russet formation is a result of extreme cuticle failure, this visual analysis fails to identify cuticles that are improperly formed, but not compromised severely enough to result in russeting. Consequently, in order to obtain a more precise characterization of fruit cuticle performance, a mechanical test was carried out. The tensile analysis performed making use of a texture analyser generated mechanical profiles that highlighted the mechanical strength and tensile progression until the breaking point (Fig. 1; Supplementary Fig. S2 at *JXB* online).

**Fig. 2.**
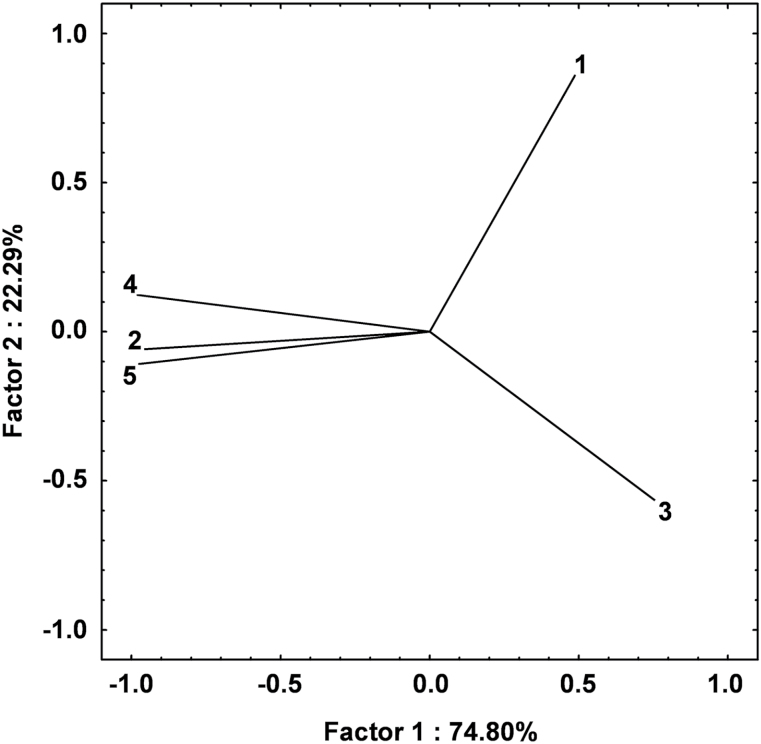
Principle component analysis loadings of mechanical traits. The projection of five parameters identified during tensile testing of the skin tissues over the hyperspace defined by PC1 and PC2 is illustrated. The variables are indicated with a numerical code: 1, gradient; 2, maximum force; 3, maximum force distance; 4, area; 5, linear distance. See Supplementary Fig. S2 at *JXB* online for more detail on how each parameter is determined.

The digital analysis of each tensile mechanical pattern led to the identification of five main parameters, considered in this study as novel descriptors for the characterization of fruit skin behaviour. The projection of these five parameters in a two-dimensional (2D)-principal component analysis (PCA) hyperspace (Fig. 2) defined by the first two principal components (explaining in total 97.09% of the phenotypic variance: PC1, 74.80%; and PC2, 22.29%) revealed their effect in the determination of the cuticle mechanical properties. The loading plot of parameters indicated that area, maximum force, and linear distance were commonly projected towards PC1, therefore playing a similar role in the explanation of the total phenotypic variance. On the other hand, the other two variables (gradient and maximum force distance) were oppositely oriented in the other two quadrants of the 2D-PCA plot (along the PC2), explaining a different portion of the total variance. The employment of these variables generated a wide data distribution, which, in general, followed a normal distribution, with the exception of maximum force and area, characterized by a more skewed type of trait segregation. The general distribution of data, with respect to the position of the two parental cultivars, suggested a transgressive type of segregation, with the seedlings exceeding the value of the two parents (Supplementary Fig. S3 at *JXB* online). Generally, the mechanical data indicated that ‘Golden Delicious’ displays a slightly higher cuticular performance in terms of strength than ‘Braeburn’. Among the indexes, the gradient was found to be the mechanical parameter most statistically correlated with the visually inspected russeting phenotype (*r*=0.35, *P*-value ≤0.05).

### QTL mapping identifies two regions linked to cuticle formation

**Fig. 3.**
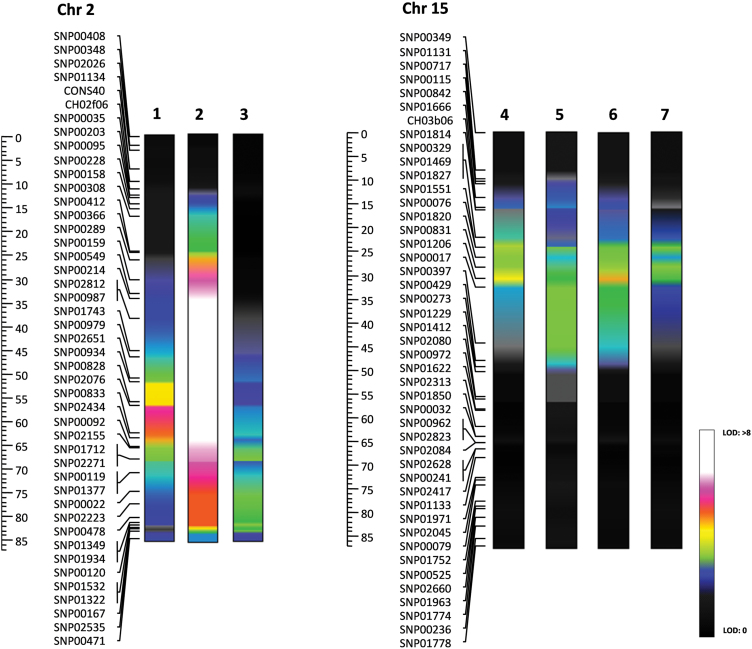
Heat map pattern of LODs for the QTL intervals detected for cuticle performance and russeting on chromosomes 2 and 15. The traits are indicated according to a numerical code as follows: 1, russeting; 2, percentage russeting; 3, gradient; 4, maximum force, 5, maximum force distance; 6, area; 7, linear distance.

The molecular map of the ‘G×B’ progeny was represented by 605 markers, uniformly distributed over 17 chromosomes (haploid number for apple), covering a total length of 1195.69 cM, with an averaged distance between adjacent markers of ~2 cM. This map was finally employed in a QTL mapping survey to identify putative genomic regions involved in the genetic control of fruit cuticle performance and the potential for russeting development. As phenotypic data, the five mechanical parameters, as well as the visual scoring of russeting (as the absence/presence of russeting and its coverage on the skin surface) were considered. By the joint analysis of these data sets, seven major QTL intervals were identified within the ‘G×B’ genome, three located on chromosome 2 and four on chromosome 15 (Fig. 3; Table 2; Supplementary Fig. S4 at *JXB* online).

**Table 2.** Details of the QTLs associated with visual inspection of russeting as well as cuticle mechanical parameters

LG	Trait	Marker	cM	LOD	%exp
2	Russet	SNP02076	56.60	6.91	30.3
	% Russeting	SNP02076	56.60	13.02	49.4
	Gradient	SNP01349	81.20	4.8	30.4
15	Maximum force	SNP01551	24	6.26	37.7
	Max force distance	SNP01206	30.7	4.41	28.3
	Area	SNP01551	24	6.41	38.4
	Linear distance	SNP01551	24	5.16	32.3

For each trait, the linkage group, the marker coincident with the highest LOD score, and the expressed variance are reported.

The first QTL identified on chromosome 2 was related to the gradient, showing a maximum LOD value of 4.8 (30% of expressed variance) and located towards the end of the linkage group (81.2 cM). The second cluster of QTLs, positioned on the same linkage group, was instead related to the russeting phenotype. QTLs related to both russeting and percentage russeting were characterized by a similar LOD shape (Supplementary Fig. S4 at *JXB* online), but with a different magnitude. The percentage of russeting which showed the greatest variance was associated with a QTL with a LOD value of 13.02 (49.4% of expressed variance), higher with respect to the qualitative evaluation (presence/absence) of russeting (LOD value of 6.91, 30.3% of phenotypic variance explained). These two QTLs were located on linkage group 2, at 56.6 cM from the top of the chromosome (Fig. 3).

The second cluster of QTLs, more related to mechanical behaviour parameters, was instead located on chromosome 15. These genomic regions were associated with the four mechanical parameters, digitally obtained from the tensile profile generated by the texture analyser during the assessment of the skin tissue of the fruit collected from the ‘G×B’ population. These QTLs, related to maximum force, maximum force distance, area, and linear distance, spanned from a LOD value of 4.41 to 6.41 (Fig. 3; Supplementary Fig. S4 at *JXB* online), with a corresponding range of phenotypic variance from 28.3% to 38.4% (Table 2). The estimated mean of the distribution associated with each of the four genotypic classes was calculated for the QTL clusters located on chromosomes 2 and 15. In both chromosomes the targeted QTLs seemed to be determined by a specific allelic combination. In chromosome 2, the QTL for both russeting and gradient was associated with the ‘bd’ allelic configuration (Supplementary Fig. S5A), while in chromosome 15 the QTL was determined by ‘ad’ Supplementary Fig. S5B). It is worth noting that in both chromosomes the allele ‘d’ of ‘Braeburn’ is associated with the presence of the QTL, and this is the apple cultivar known to transmit the russeting trait when used as the parental genotype in controlled crossing (Wood, 2001).

### Expression profiling of candidate genes in selected progeny links the AP2-type MdSHN3 transcription factor with proper cuticle formation

To better investigate the genetic mechanisms underlying the control of fruit cuticle properties in apple, an *in silico* gene mining was performed by anchoring the QTL genomic intervals targeted on chromosomes 2 and 15 on the assembled version of the ‘Golden Delicious’ apple genome (Velasco *et al.*, 2010), and identifying a total of 3841 and 1675 predicted genes, respectively (Supplementary Tables S2, S3 at *JXB* online). Through manual inspection, within these two gene lists, a set of candidates was identified based on similarity to previously characterized cuticle-related genes from other plant species. The full list of candidate cuticle genes together with their similarity to characterized orthologues is provided in Table 3.

**Table 3.** Genes identified in the QTL regions putatively linked to cuticle biosynthesis or regulation

Gene ID	Ch.	Name	Description/role	Orthologue ID	Homology (idendities/ positives)	Reference
MDP0000287191	2	*WSD11*	Wax ester synthase/ diacylglycerol acyltransferase	AT5G53390	31%/50%	Takeda *et al.* (2013)
MDP0000250127	2	*KAS1*	Crucial for fatty acid synthesis	AT5G46290	74%/84%	Wu and Xue (2010)
MDP0000069348	2	*CER1*	Likely aldehyde decarbonylase involved in wax synthesis	AT1G02205	57%/72%	Bourdenx *et al.* (2011)
MDP0000297929	2	*DGAT1*	Lipid biosynthesis	AT2G19450	74%/83%	Zhang *et al.* (2009)
MDP0000938736	15	*KCS11*	Biosynthesis of very long chain fatty acids	AT2G26640	79%/88%	Blacklock and Jaworski (2006); Li-Beisson *et al.* (2013)
MDP0000178263	15	*SHN3*	Transcriptional regulator of cuticle biosynthesis	AT5G25390	58%/68%	Aharoni *et al.* (2004)
MDP0000729533	2	*BAHD ACYL-TRANSFERASE*	Transferring acyl groups other than amino-acyl groups	AT3G26040	40%/58%	Kosma *et al.* (2012; Li-Beisson *et al.* (2013)
MDP0000391122	2	*BAHD ACYL-TRANSFERASE*	Transferring acyl groups other than amino-acyl groups	AT3G26040	38%/54%	Kosma *et al.* (2012); Li-Beisson *et al.* (2013)

*CER1*, *ECERIFERUM1*; *DGAT1*, *DIACYLGLYCEROL O-ACYLTRANSFERASE1*; *KAS1*, *KETOACYL-ACP SYNTHASE1*; *KCS11*, *3-KETOACYL-COA SYNTHASE11*; *WSD11*, *WAX ESTER SYNTHASE/DIACYLGLYCEROL ACYLTRANSFERASE11*.

**Fig. 4.**
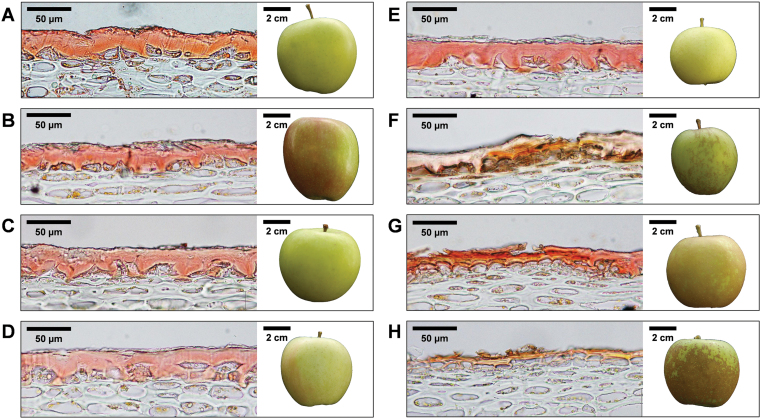
Histological analysis of apple skin tissues in a selected subset of the population. Light microscopy analysis of skin samples stained with Sudan IV are shown, together with photographs of whole apples. Sudan IV stains the cuticular lipids a pink/orange colour. (A, B) Parental lines ‘Golden Delicious’ (A) and ‘Braeburn’ (B), show regular cuticles. (C–E) Selected progeny with regular skin are shown: (C) G×B 3; (D) G×B 6; (E) G×B 11. (F–H) Selected progeny with compromised cuticles are shown: (F) G×B 30; (G) G×B 59; (H) G×B 90. Thinner, cracked cuticles are clearly visible in these lines, whereas whole apple photographs show the presence of russet on the surface of the fruit.

Three seedlings showing compromised cuticle structure were selected based on prior tensile testing as well as the appearance of russeting (G×B 30, G×B 59, and G×B 90), and three seedlings with regular skin (G×B 3, G×B 6, and G×B 11). These lines were used together with both parental cultivars for more detailed investigation. Light microscopy on cross-sections of skin tissues confirmed a correlation between the tensile tests (particularly the maximum force parameter) and cuticle formation (Fig. 4; Supplementary Fig. S6 at *JXB* online). Lines with fruit skin requiring less force to break during the tensile testing showed dramatically thinner and sometimes cracked cuticles when compared with the parents as well as with the progeny identified as displaying regular cuticle performance during the mechanical testing (Fig. 4). While russet was not necessarily observed to cover the entire fruit (as seen in the fruit image in Fig. 4F), microscopy showed that in compromised cuticle lines even in areas where the production of russet was absent, the cuticle was dramatically thinner (Fig. 4F).

**Fig. 5.**
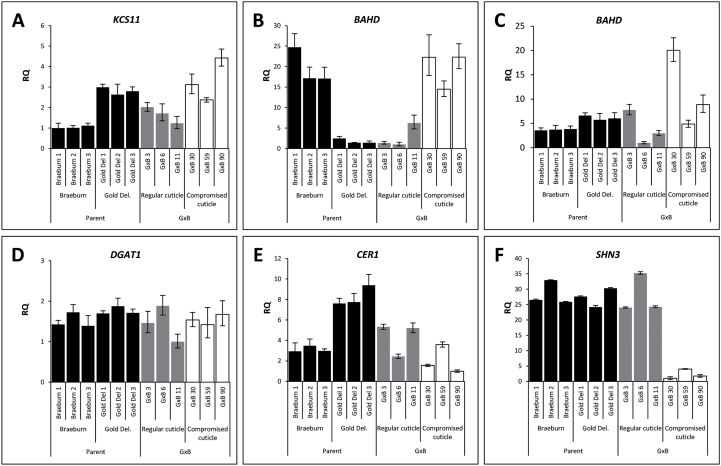
Expression analysis of candidate genes in early green stage peel tissue. Determination of the expression of candidate genes was performed via quantitative real-time PCR. (A) *KCS11*, *3-KETOACYL-COA SYNTHASE11*; (B) *BAHD ACYL-TRANSFERASE* (*MDP0000391122*); (C) *BAHD ACYL-TRANSFERASE* (*MDP0000729533*); (D) *DGAT1*, *DIACYLGLYCEROL O-ACYLTRANSFERASE1*; (E) *CER1*, *ECERIFERUM1*; (F) *SHN3*. Parent lines are shown in black, progeny determined to possess regular cuticle are grey, and progeny displaying compromised cuticles are white. Error bars show the standard error (*n*=3).

**Fig. 6.**
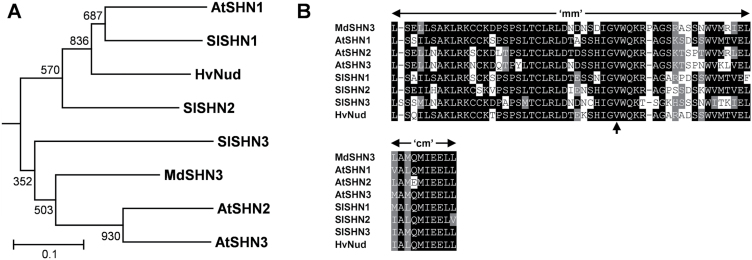
Molecular phylogeny and sequence analysis of MdSHN3. (A) Molecular phylogeny of characterized members of the SHN1/WIN1 clade transcription factors shows MdSHN3 to be a member of this clade. (B) Alignments of two domains shown to be functionally relevant to this clade are displayed, with the functionally critical valine residue indicated with an arrow. Alignments were performed using Clustal Omega, and Neighbor–Joining phylogenetic trees constructed with MEGA 5.0. Values at branch points are bootstrap values calculated from 1000 replicates. AtMYB9 was used as an outlier to create a root for tree and is not shown. ‘mm’, middle motif; ‘cm’, C-terminal motif.

Expression analysis was performed on candidate genes in the selected progeny and parents to identify a potential link between the candidates and the observed cuticular phenotypes. The early green stage of fruit development was selected for expression analysis, as already at this stage early signs of russet formation could be seen in selected progeny. Of the eight genes (located within the QTLs mapped on chromosomes 2 and 15) identified as potential candidates, expression was detected for six (Fig. 5). No expression was detected in any of the samples for the *WAX ESTER SYNTHASE/DIACYLGLYCEROL ACYLTRANSFERASE* (*WSD11*, *MDP0000287191*) and *KETOACYL-ACP SYNTHASE1* (*KAS1*, *MDP0000250127*) orthologues. Only one candidate gene showed a significant segregation with the compromised cuticle phenotype, *MDP0000178263* (*MdSHN3*) (Fig. 5F). Sequence analysis and construction of a phylogenetic tree revealed that *MdSHN3* is an orthologue of the cuticle-associated *SHN1/WIN1* clade of AP2-domain transcription factor genes (Fig. 6). *MdSHN3* was expressed at relatively similar levels across the parental cultivars, and the three selected progeny showing regular cuticles; however, in the three selected progeny showing compromised cuticles, *MdSHN3* was expressed up to 25-fold less. Expression analysis for *MdSHN3* was extended to include the mature green and harvest stage, showing that expression levels for *MdSHN3* remain low in the lines with compromised cuticles throughout development (Supplementary Fig. S7 at *JXB* online). A 70-fold decrease in *MdSHN3* expression was observed at the mature green stage for lines with compromised cuticles, while the difference at the harvest stage was lower (25-fold; Supplementary Fig. S7).

Finally, it is interesting to note the expression pattern of *MDP0000391122* (a *BAHD acyltransferase*), which shows similar expression levels in the parental cultivar ‘Braeburn’, as well as in the progeny lines with compromised cuticles, while expression in the parental cultivar ‘Golden Delicious’, and the seedlings with regular cuticles show a 15-fold decrease in expression (Fig. 5B).

## Discussion

### Use of five novel mechanical parameters allows for a high-throughput analytical characterization of apple cuticle performance

The plant cuticle plays a vital role in the protection of whole organs and especially fruit, preserving the integrity and quality during development and also during post-harvest storage (Albert *et al.*, 2013). In apple, the formation of russet is caused directly by cuticle failure. In an effort to identify QTL regions linked to this disorder, the visible russet formation in the ‘G×B’ progeny as well as the mechanical properties of the fruit skin tissue in this population were characterized. Russet can be considered a secondary effect due to cuticle failure, and is not necessarily present in fruit displaying mildly compromised cuticles. This was confirmed by microscopic observations showing dramatically thinner cuticles in lines with compromised cuticle formation yet lacking extensive russet formation (Fig. 4). Analysing the mechanical properties of the cuticles therefore allowed targeting of the potential cause of the russet seen in the population. This was accomplished using an instrument previously employed for the dissection of the apple fruit texture complexity (Longhi *et al.*, 2012). By the controlled tension applied on the skin portion isolated from the apple of the ‘G×B’ progeny, five parameters were derived, which were eventually implemented as quantitative traits for a specific and detailed QTL mapping assay. In fact, these parameters allow the characterization of a continuous trait distribution, which is a fundamental requirement for QTL computation. Among the five parameters, area, maximum force, and linear distance were commonly oriented over the 2D-PCA plot, and can be considered as describing cuticle strength, a trait specifically associated with the cutin matrix (Pollard *et al.*, 2008; Schreiber, 2010). The other two variables, maximum force distance and gradient, displayed different projections. The maximum force distance, which showed an almost opposite orientation with regards to the first set of parameters, is probably also associated with the mechanical strength of the skin. An interesting observation is the orthogonal projection of the gradient parameter, explaining novel phenotypic variance when compared with the other variables measured. This parameter is related to the skin elasticity properties, rather than to the strength of the skin, and correlates most strongly with the visually observed russeting phenotype (Fig. 2). Russeted skin tissue is known to have an altered elasticity when compared with regular peels (Khanal *et al.*, 2013). These parameters can therefore be used for a more specific and analytical characterization of the fruit cuticle performance and subsequent russeting in a reliable and high-throughput fashion. The development of these novel parameters allowed, in fact, a more precise, comprehensive, and reliable characterization of the peel behaviour in apple, further validated by the QTL mapping survey. The detection of two QTL regions specifically associated with these parameters (one in common with the russeting visually scored) confirms their efficiency in describing this particular phenotype.

### QTLs on chromosomes 2 and 15 are associated with cuticle formation and subsequent russeting

The implementation of the mechanical parameters and the russeting evaluation, together with the genotypic data, allowed the identification of a set of QTLs on two specific chromosomes, 2 and 15. The QTLs identified on chromosome 2 were associated with the visual inspection for russeting and the gradient parameter (describing elasticity) of the tensile profile. This supports the link between russet formation and a modulation in the elasticity of the cuticle. Regarding the estimation of russeting, the highest LOD value was detected for the evaluation of the percentage of corky tissue over the fruit surface, validating the fact that a quantitative type of trait provides more reliable and precise data than a qualitative scoring for QTL investigation. This may also be the reason for the different resolution and position (although overlaying) for the QTL detected for the gradient parameter (Fig. 3; Supplementary Fig. S4 at *JXB* online).

The other measured tensile parameters (maximum force, area linear distance, and maximum force distance) were associated with a QTL region on chromosome 15 (Fig. 3). These parameters can all be thought to describe the strength of the cuticle and all predicted highly similar QTL LOD profiles. These parameters are able to identify weaker cuticles that may not have been compromised to the extent to which they develop russet. This is vitally important when investigating the primary cause of russet formation.

It is interesting to note that chromosomes 2 and 15 arose from a single ancestral chromosome during the recent genome duplication event in apple which resulted in several chromosome pairings (Velasco *et al.*, 2010). The involvement of more than one chromosome in the control of cuticle biosynthesis in fleshy fruits is neither novel nor surprising, as demonstrated by the widespread localization of genes described in tomato fruit cuticle biosynthesis (Hen-Avivi *et al.*, 2014; Martin and Rose, 2014). Whole-genome duplication events, however, can allow species to generate diversification and novelties within its metabolism (Mühlhausen and Kallmar, 2013). For example, the sequencing of the tomato genome (Tomato Genome Consortium, 2012) revealed that genome duplication events probably determined the subfunctionalization of new gene family members, crucial for the establishment of fruit-specific functions, including ripening. In the case of this study, it appears that the QTL identified on chromosome 15 can be thought of as primary cause of cuticle failure, while the QTL on chromosome 2 is possibly related to enhancing the subsequent production of russeted tissue.

The analysis of the effect of each QTL detected over the ‘G×B’ genome highlighted in this particular genetic background that cuticular performance, as well as the russeting trait, was under a recessive genetic control. By plotting the estimated mean of the distribution associated with each of the four genotypic classes, it can be seen that the trait was associated with the combination of two specific alleles. Between the two varieties, moreover, the allele ‘d’ of ‘Braeburn’ was the one required for the expression of the phenotype in both cases: the occurrence of russeting on chromosome 2 and the cuticle mechanical performance on chromosome 15. This finding validates the hypothesis of a recessive genetic control, as initially suggested by the segregation ratio of 3:1, following the observation that ~20% of the progeny showed a russeting phenotype. The slightly lower incidence of observed russeting than would be expected in a 3:1 segregation can be explained by the fact that the russet is a secondary consequence of cuticle failure, which does not always result in russet formation.

The markers identified in correspondence with the highest LOD value for both QTL regions may be exploited in the future as important tools in marker-assisted programmes for the achievement of two important goals. First of all, the marker associated with the QTL on chromosome 2 can be useful for the advanced selection of those seedlings which have inherited the alleles associated with russeting. While this trait is considered valuable in some heirloom and traditional varieties (such as ‘Renetta Canada’, ‘Renetta Grigia’, or ‘Tyroler Spitzlederer’), it is typically an undesirable trait, due for both consumer preference and long-term storage ability. In the present study it was demonstrated that russeting can occur in progeny arising from crossing of two regular skin varieties (i.e. ‘Golden Delicious’ and ‘Braeburn’) at a relatively high rate. It would therefore be advantageous to screen for this phenotype at an early stage.

The identification of the QTL on chromosome 15 associated with cuticle performance can be employed to select novel accessions characterized by better performing cuticles. Taking into consideration that the horticultural management and production system for apple post-harvest storage is an essential step to guarantee its marketability, a high performing cuticle is a fundamental requirement to guarantee and maintain favourable fruit quality. Furthermore, as russeting is a secondary effect of cuticle failure, one would also be selecting against potential russet formation.

### MdSHN3 promotes cuticle biosynthesis and prevents russet in apple

Analysis of the two chromosomal regions identified via QTL analysis revealed eight potential genes involved in cuticle biosynthesis and regulation. These genes included an orthologue to *AtWSD11* on chromosome 2, shown previously to be involved in wax ester synthesis in *Arabidopsis*, where it is required to prevent organ fusions caused by a malformed cuticle (Takeda *et al.*, 2013). An orthologue to the fatty acid synthesis gene *AtKAS1* (Wu and Xue, 2010) was also identified in the QTL on chromosome 2, as well as an orthologue to *AtCER1*, an aldehyde decarbonylase (Bourdenx *et al.*, 2011). This latter gene has been shown to be involved in cuticle wax synthesis in *Arabidopsis* and cucumber (Bourdenx *et al.*, 2011; Bernard *et al.*, 2012; Wang *et al.*, 2015). Finally, also on chromosome 2, an orthologue to the lipid biosynthesis *AtDGAT1* gene (Zhang *et al.*, 2009) was identified, as well as two BAHD acyl-transferase genes. This class of BAHD acyl-transferases have been shown to be involved in cutin and suberin polymer biosynthesis (Panikashvili *et al.*, 2009; Kosma *et al.*, 2012; Molina and Kosma, 2015). Two further genes were identified in the QTL region on chromosome 15, including an orthologue of the *Arabidopsis 3-KETOACYL-COA SYNTHASE11* (*AtKCS11*), a gene involved in the biosynthesis of very long chain fatty acids (Blacklock and Jaworski, 2006), and *MdSHN3*, an AP2-domain transcription factor gene belonging to the *SHN1*/*WIN1* clade (Aharoni *et al.*, 2004; Broun *et al.*, 2004; Shi *et al.*, 2013).

In order to determine the expression profile of these genes in progeny with a range of cuticle phenotypes, a subset was selected. This subset was represented by three progeny with regular peels, and three with compromised cuticles (as determined by tensile testing) as well as the two parental lines (Fig. 4). Deficient cuticle formation was confirmed via histological analysis, showing a dramatic reduction in the normally thick apple cuticles in the progeny of lines with compromised cuticles. This confirmation via histological analysis validated the mechanical testing performed on the whole population and illustrated the damage caused by cuticle reduction and russet formation. Expression analysis of the eight genes in this selected subset of lines revealed only the expression of *MdSHN3* to segregate exclusively with the various phenotypes associated with an improperly formed cuticle (Fig. 5F). Specifically, *MdSHN3* showed a decrease in expression between 25- and 70-fold in the progeny with compromised cuticles depending on developmental stage.

Another gene showing an interesting expression profile is the BAHD acyl-transferase gene (*MDP0000391122*). The expression profile of this gene is significantly higher in the three seedlings with compromised cuticles, as well as the parental line ‘Braeburn’ (Fig. 5B). Although this expression pattern does not segregate with the cuticle phenotype, it may indicate a role for this gene in the secondary formation of russet. BAHD acyl-transferases have in fact been implicated in suberin biosynthesis (Kosma *et al.*, 2012; Molina and Kosma, 2015) and, as ‘Braeburn’ is the parental line thought to be responsible for genetically transmitting this phenomenon, it is conceivable that *MDP0000391122* plays a role in suberin biosynthesis in lines with compromised cuticles.

The *SHN1*/*WIN1* transcription factor gene displaying an expression pattern segregating with the compromised cuticle phenotype is a strong candidate to be the underlying factor of the russeting phenotype. *SHN* transcription factor genes have previously been identified and characterized in *Arabidopsis*, tomato, and barley (Aharoni *et al.*, 2004; Taketa *et al.*, 2008; Shi *et al.*, 2013). In both *Arabidopsis* and tomato, characterized orthologues control cuticle deposition as part of a transcriptional network of epidermal cell differentiation (Aharoni *et al.*, 2004; Shi *et al.*, 2011, 2013). Transgenic lines in tomato with reduced expression of *SlSHN3* showed a severe disruption of normal fruit cuticle deposition, which resulted in an increase in both susceptibility to fungal infection and post-harvest water loss (Shi *et al.*, 2013; Buxdorf *et al.*, 2014). On the other hand, overexpression of *AtSHN1* in *Arabidopsis* resulted in increased drought tolerance and recovery (Aharoni *et al.*, 2004). In barley, an SHN orthologue, HvNud, regulates the biosynthesis of lipids which coat the caryopses (dry fruit of barley) (Taketa *et al.*, 2008). This lipid coverage of barley grains can be seen as analogous to the cuticle of fleshy fruit.

Sequence analysis of the MdSHN3 protein confirmed the presence of the AP2 domain, characteristic of the larger family, and also the two conserved motifs characteristic of the SHN1/WIN1 subclade (Fig. 6B). The so-called ‘middle motif’ (‘mm’) and the ‘C-terminal motif’ (‘cm’) have been recognized as important regulatory domains for this clade of transcription factors (Aharoni *et al.*, 2004). Furthermore, the conserved valine residue in the ‘mm’ identified by Taketa *et al.* (2008) and demonstrated to be critical for SHN enzyme function is also present in the MdSHN3 protein (Fig. 6B).

Taken together, the expression pattern, the identification of conserved functional protein domains, and the genomic location within the high confidence interval of a QTL linked strongly to skin strength show that it is likely that in apple fruit *MdSHN3* positively regulates cuticle deposition and subsequent thickness. This might occur via the transcriptional control of downstream cuticle biosynthesis target genes, as was previously demonstrated in tomato (Shi *et al.*, 2013). Expression of *MdSHN3* throughout apple fruit development (as shown to be the case in fruit with regular skin; Supplementary Fig. S7 at *JXB* online) is therefore crucial to promote proper cuticle formation. The histological analysis of the fruit skin of the progeny with decreased *MdSHN3* expression illustrates the major reduction in cuticle formation in these lines, which subsequently resulted in diminished peel strength, and an increased potential for russet development.

## Conclusions

The simultaneous investigation of the tensile performance of apple cuticles and the occurrence of russeting demonstrated the relationship between these two properties, i.e. cuticle failure is a prerequisite for apple russet formation. These parameters, together with the markers identified within the QTLs detected over two chromosomes, can be considered as a valuable tool in assisted breeding programmes, in order to predict the development of this undesirable trait in seedlings. The recent advances in the understanding of cuticle biology in model species such as *Arabidopsis* and tomato can now be applied to agriculturally important crops. In this case, prior knowledge was expanded and evidence was provided that an apple orthologue of *SHN3* positively regulates cuticle biosynthesis, thus preventing apple russet. This knowledge in terms of apple production is invaluable when one considers the economic importance of a long post-harvest storage for apple fruit. The identification of a functional orthologue of the SHN1/WIN1 clade of transcription factors in apple further highlights the importance of this clade of transcription factors as primary regulators of epidermal processes, particularly cuticle biosynthesis.

## Supplementary data

Supplementary data are available at *JXB* online.

Figure S1. Texture analyser device employed in this investigation, showing the clamps used to hold the peel for the tensile analysis.

Figure S2. Mechanical tensile profile, with the five parameters identified by the macro highlighted.

Figure S3. Trait distribution over the ‘G×B’ progeny.

Figure S4. LOD profile for the QTLs associated with the two indexes used to estimate the russeting development (Russet and % Russet), as well as the five tensile parameters.

Figure S5. Estimated mean of the total distribution associated with each genotype class.

Figure S6. Force required to break peels.

Figure S7. Developmental gene expression analysis of *MdSHN3* in three specific stages of fruit development.

Table S1. Oligonucleotides used in this study.

Table S2. List of genes annotated within the QTL interval identified on chromosome 2.

Table S3. List of genes annotated within the QTL interval identified on chromosome 15.

Supplementary Data
