# Supplementary material for: Genome investigation suggests MdSHN3, an APETALA2-domain transcription factor gene, to be a positive regulator of apple fruit cuticle formation and an inhibitor of russet development
Source: J Exp Bot. 2015 Jul 28;66(21):6579–89. doi: 10.1093/jxb/erv366 (PMC4623677; doi:10.1093/jxb/erv366)
Supplement: Supplementary Data [file supp_66_21_6579__index.html]

Genome investigation suggests MdSHN3, an APETALA2-domain transcription factor gene, to be a positive regulator of apple fruit cuticle formation and an inhibitor of russet development — Genome investigation suggests MdSHN3, an APETALA2-domain transcription factor gene, to be a positive regulator of apple fruit cuticle formation and an inhibitor of russet development — Supplementary Data 

# Genome investigation suggests *MdSHN3*, an APETALA2-domain transcription factor gene, to be a positive regulator of apple fruit cuticle formation and an inhibitor of russet development

## Supplementary Data

Data files

- Supplementary Data - Supplementary Data
- Supplementary Data - Supplementary Data
