# Supplementary material for: Genome investigation suggests MdSHN3, an APETALA2-domain transcription factor gene, to be a positive regulator of apple fruit cuticle formation and an inhibitor of russet development
Source: J Exp Bot. 2015 Jul 28;66(21):6579–89. doi: 10.1093/jxb/erv366 (PMC4623677; doi:10.1093/jxb/erv366)
Supplement: Supplementary Data [file supp_erv366_jexbot150680_file001.pdf]

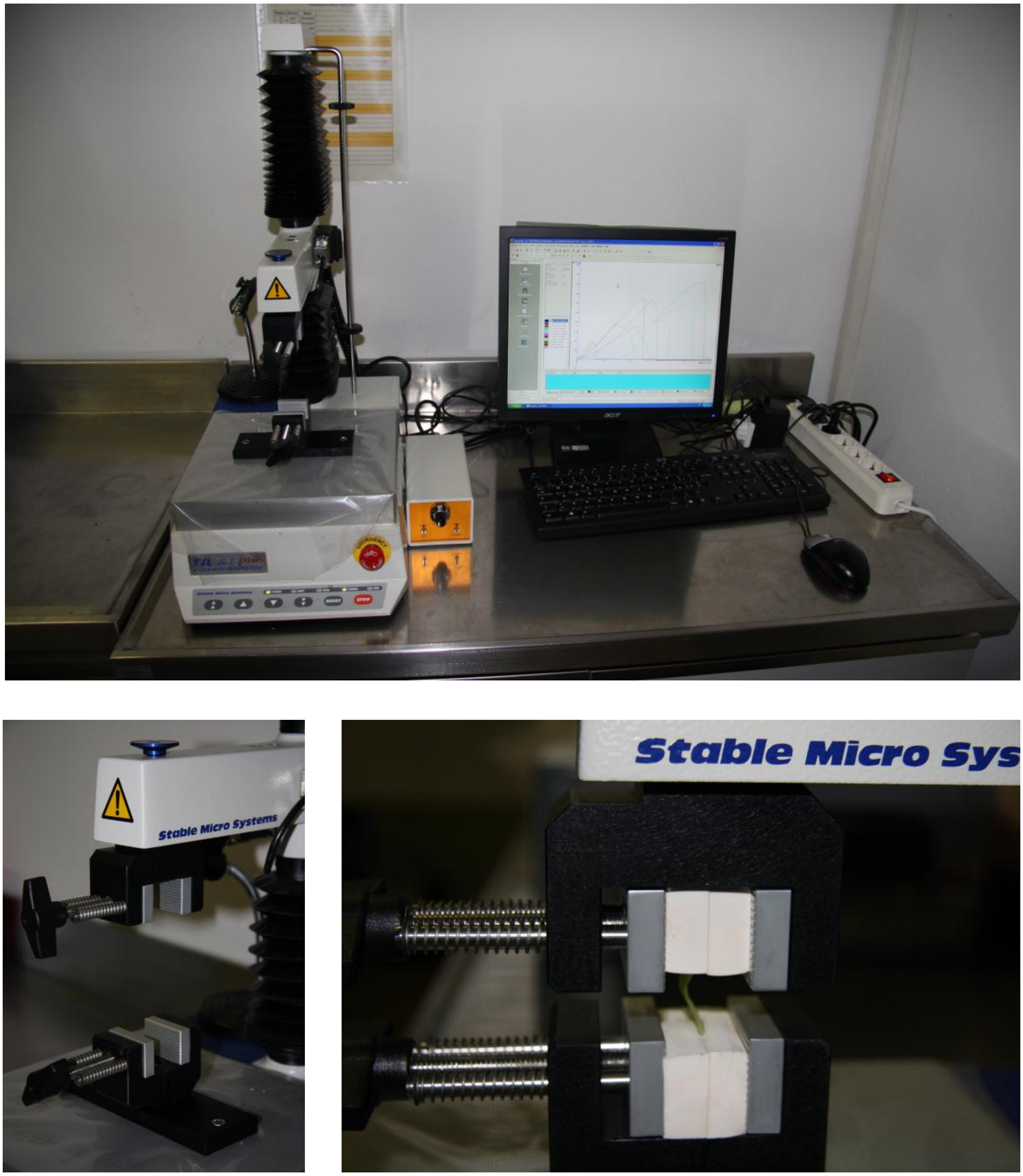

Supp. Figure 1
Lashbrooke *et al*., (2015)

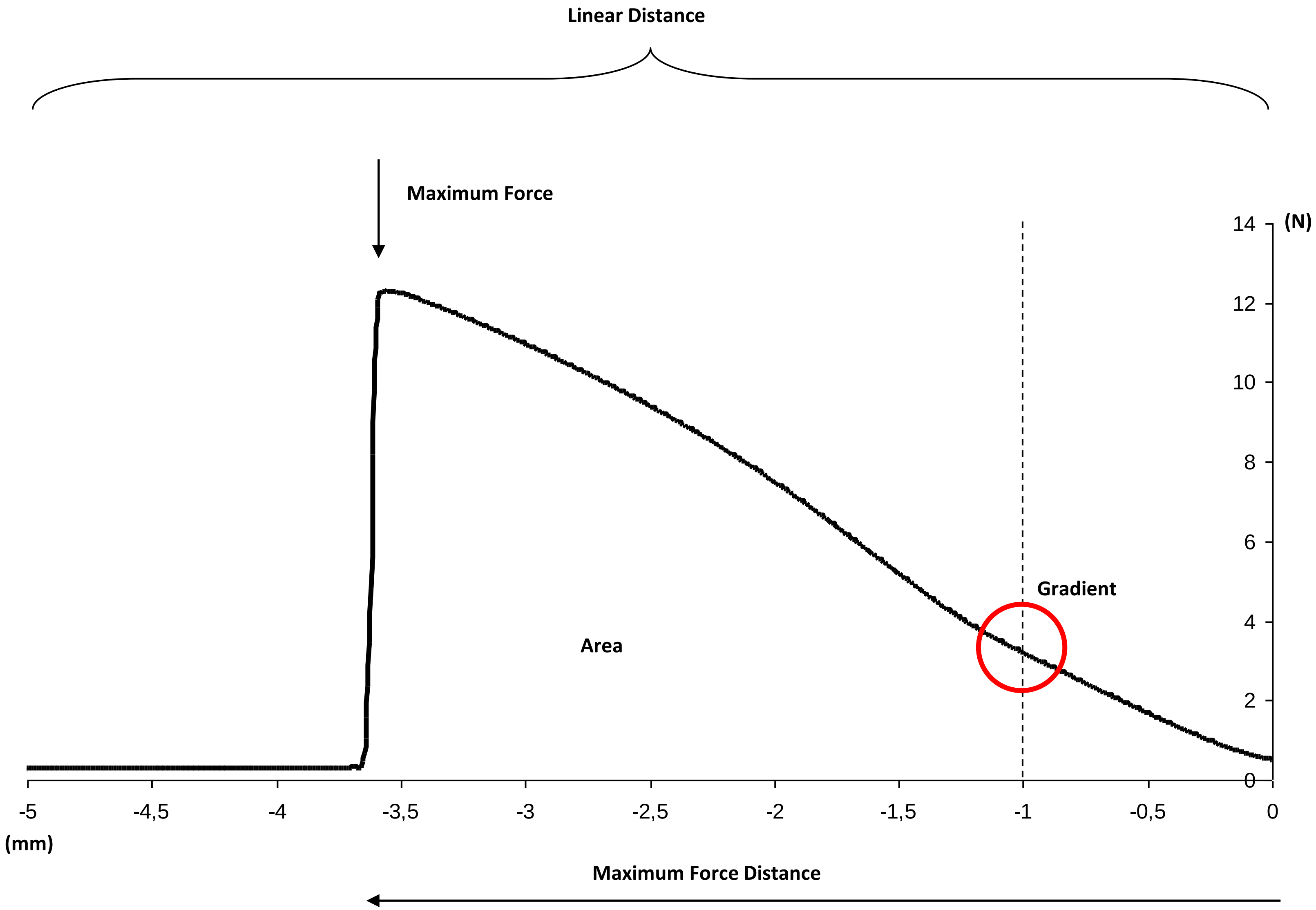

Supp. Figure 2
Lashbrooke *et al.*, (2015)

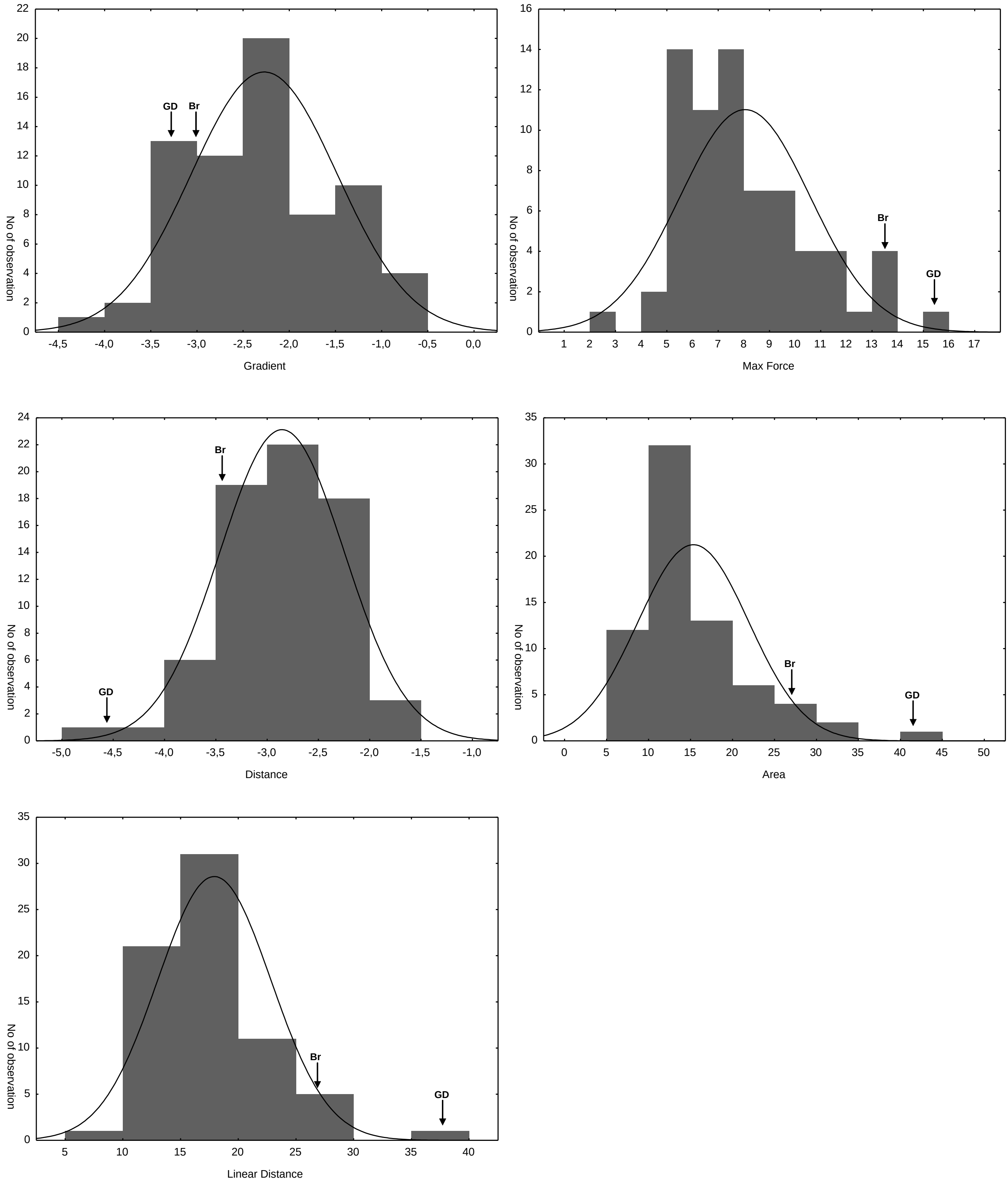

Supp. Figure 3
Lashbrooke *et al.*, (2015)

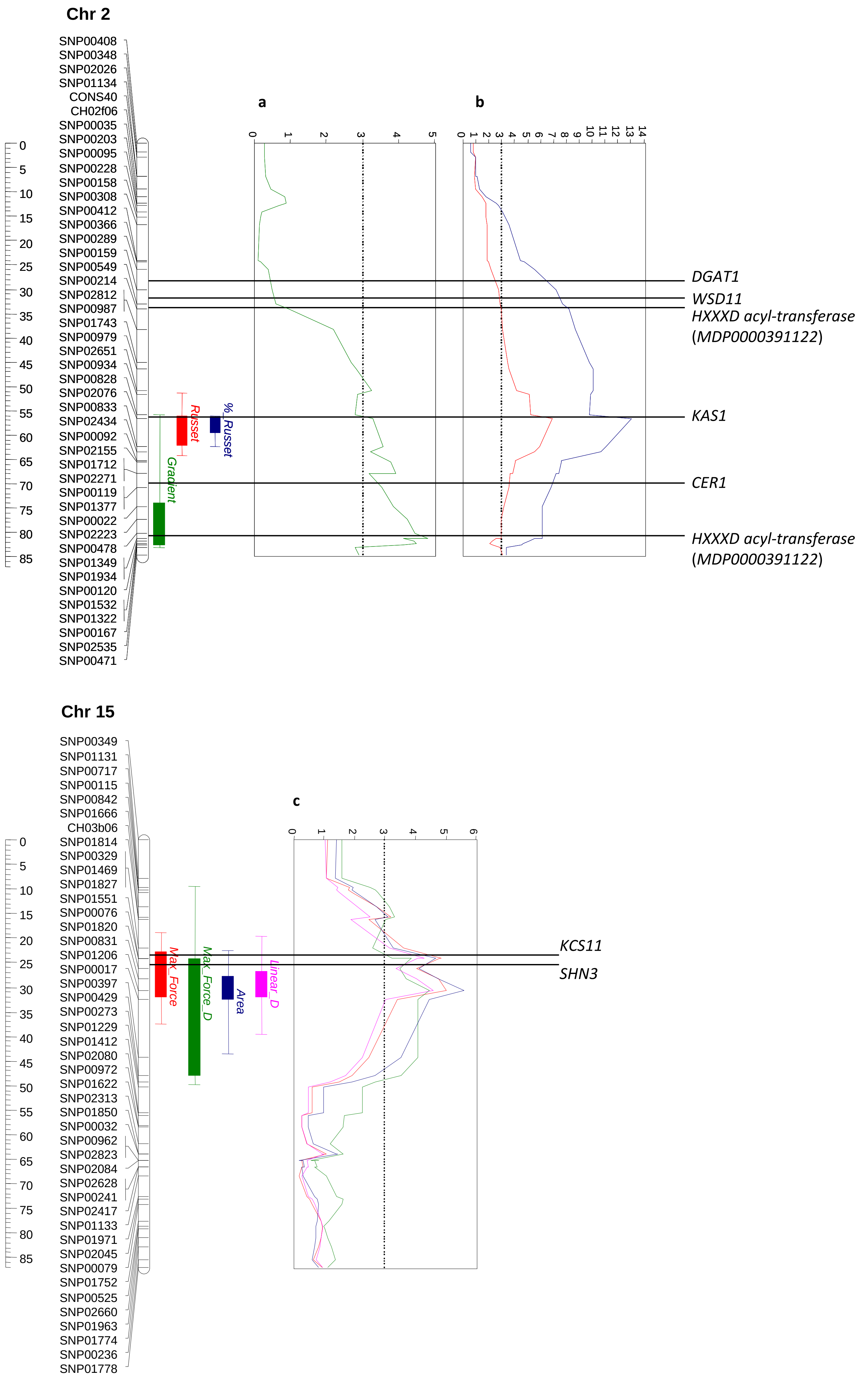

Supp. Figure 4
Lashbrooke *et al.*, (2015)

A

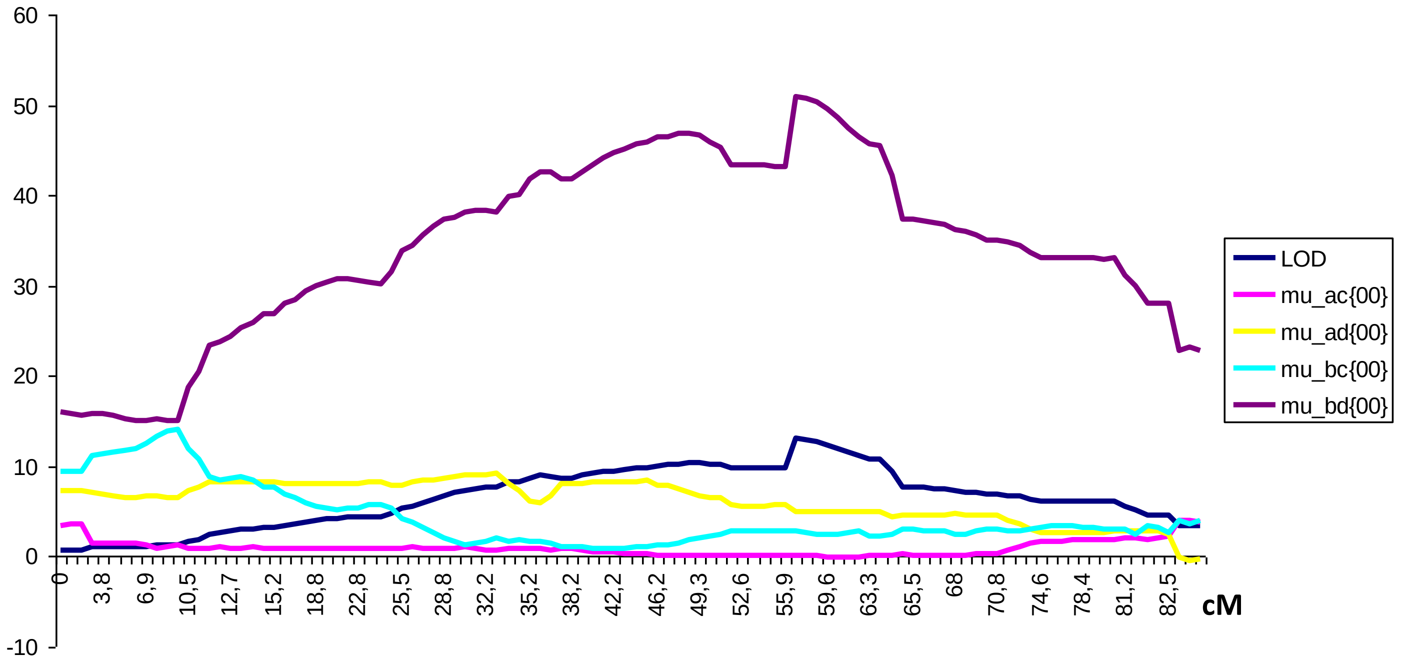

B

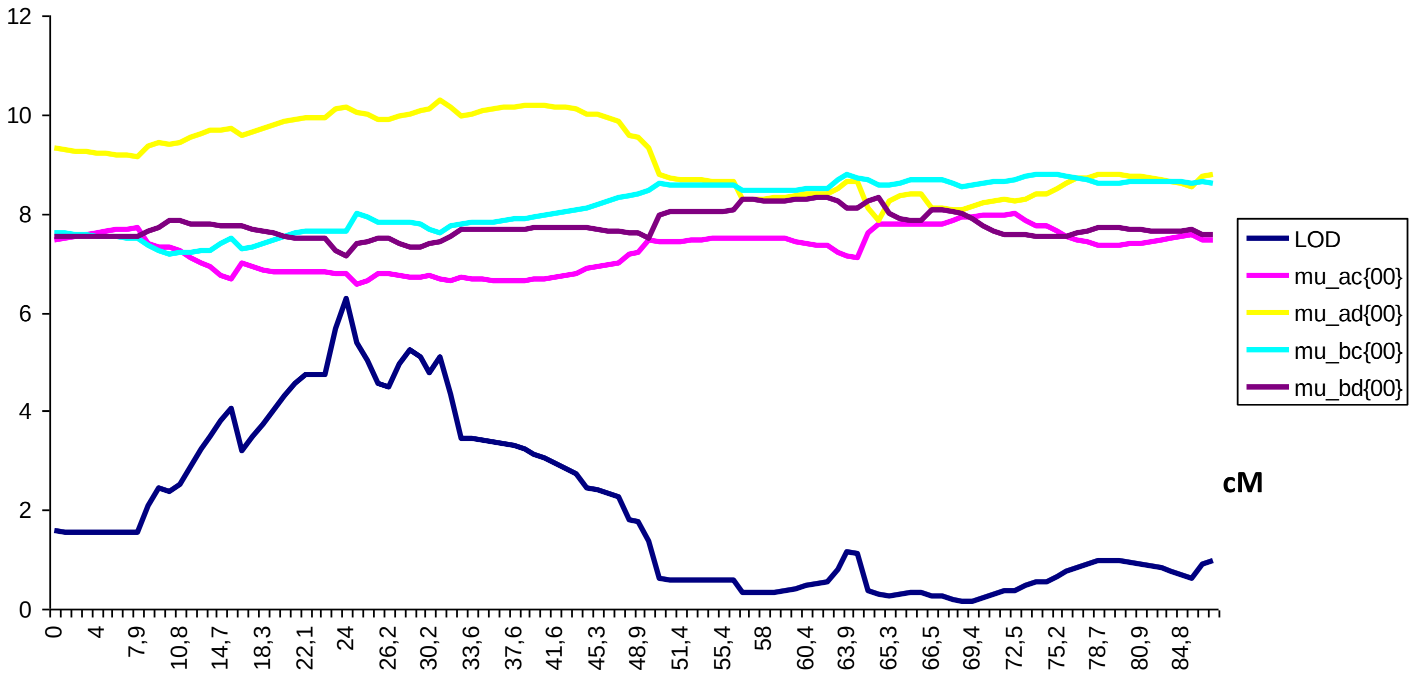

Supp. Figure 5
Lashbrooke *et al.*, (2015)

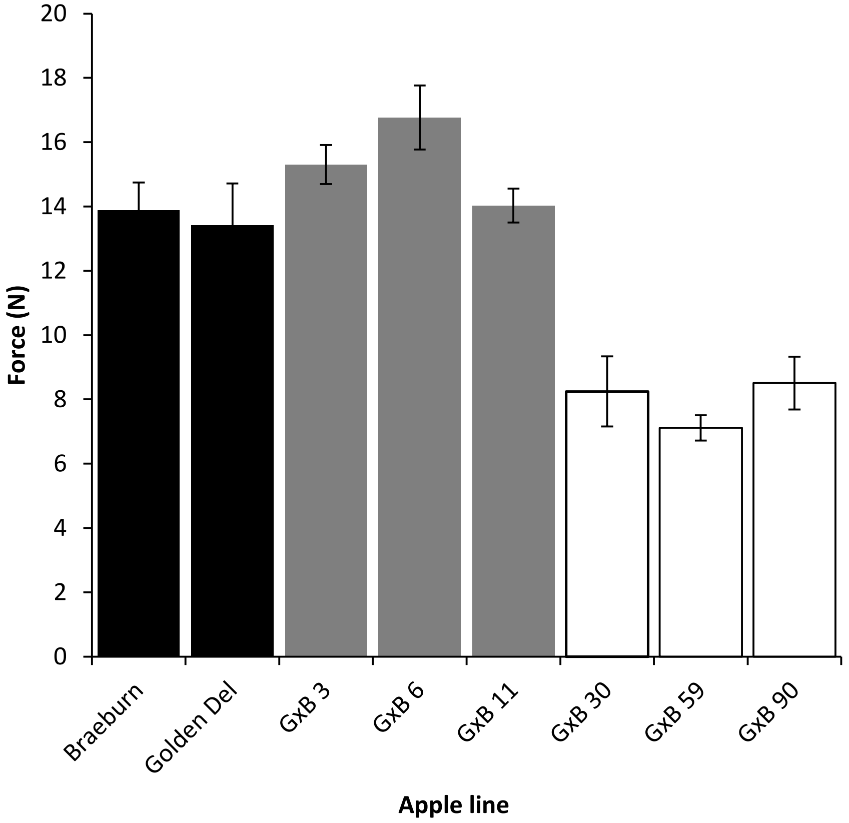

Supp. Figure 6
Lashbrooke *et al*., (2015)

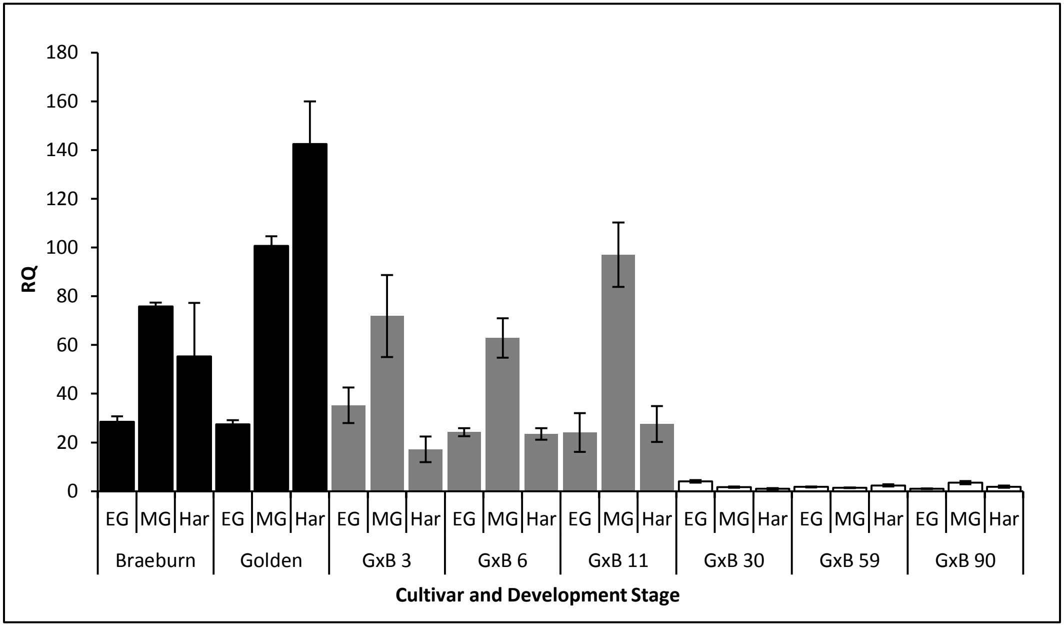

Supp. Figure 7

Lashbrooke *et al.*, (2015)

Supplementary Table 1. Oligos used in this study to measure gene expression

Gene ID	Name	Forward primer	Reverse primer
MDP0000287191	WSD11	CTCTGCAATGCATGTTCAGCAT	GGTCATGTGAGATGTTGCTTCTGT
MDP0000250127	KAS1	TGAAGAAGCAACATGAGGTTAACG	GGCTCAGTGCTCCCTTTCCT
MDP0000069348	CER1	TGATTGGCCATGGAAACCA	AATGACCCAAGGAGCCATGAT
MDP0000938736	KCS11	CTGTTATACAGGTGCCTCCCAATC	AGCTGATCAAGGGCACCAAA
MDP0000178263	SHN3	CTGCCACCGACCAAAACACT	TGGGTCTTTGCAGCATTTCC
MDP0000729533	HXXXD-type acyl-transferase	GATGTGGTGCCTCTCGTAGCA	TGGCGGTGAGTTTAGGAAATCTAG
MDP0000391122	HXXXD-type acyl-transferase	GCCAGTCAAGGATCCTCACAAG	CCCGCCACATTCAAAGAAGTT
MDP0000297929	DGAT1	GACGGCAATTCCTTGAACGA	CCGGCGTCGCAGATTTT
